# Biological Evaluation and Docking Analysis of Daturaolone as Potential Cyclooxygenase Inhibitor

**DOI:** 10.1155/2016/4098686

**Published:** 2016-03-02

**Authors:** Abdur Rauf, Francesco Maione, Ghias Uddin, Muslim Raza, Bina S. Siddiqui, Naveed Muhammad, Syed Uzair Ali Shah, Haroon Khan, Vincenzo De Feo, Nicola Mascolo

**Affiliations:** ^1^Department of Geology, University of Swabi, Khyber Pakhtunkhwa, Anbar 23561, Pakistan; ^2^Department of Pharmacy, University of Naples Federico II, 80131 Naples, Italy; ^3^Institute of Chemical Sciences, University of Peshawar, Peshawar 25120, Pakistan; ^4^H.E.J. Research Institute of Chemistry, International Center for Chemical and Biological Sciences, University of Karachi, Karachi 75270, Pakistan; ^5^Department of Pharmacy, Abdul Wali Khan University Mardan, Mardan 23200, Pakistan; ^6^Department of Pharmacy, University of Salerno, Fisciano, 84084 Salerno, Italy

## Abstract

This study deals with the isolation of the active constituent(s) from a methanolic extract of* Pistacia integerrima* J. L. Stewart barks and it was also oriented to evaluate the* in vivo* and* in silico* anti-inflammatory activity. By NMR and crystallography techniques, we have isolated a triterpenoid identified as daturaolone (compound** 1**). This compound showed* in vivo* a significant and dose dependent (1–30 mg/kg) anti-inflammatory activity on carrageenan-induced mouse paw oedema (ED_50_ = 10.1 mg/kg) and on acetic acid-induced writhing responses in mice (ED_50_ = 13.8 mg/kg). In the* in vivo* experiments, the effect of tested compound was also evaluated in presence of the reference drug diclofenac (1–30 mg/kg). Moreover,* in silico* analysis of receptor ligand complex shows that compound** 1** interacts with cyclooxygenases (COXs) binding sites displaying an interesting interaction with COX-1. These findings suggest that compound** 1** isolated from* P*.* integerrima* possesses* in vivo* anti-inflammatory and antinociceptive potentials, which are supported* in silico* by an interaction with COXs receptors.

## 1. Introduction

*Pistacia integerrima* J. L. Stewart is one of twenty species belonging to the genus* Pistacia* (Anacardiaceae). This moderate size tree widely grows in the subalpine regions of Himalaya as well as in various regions of Pakistan and India [1, 2]. Many species of genus* Pistacia* are used in traditional medicine against various ailments [3]. In fact their plant extracts have been studied for a variety of biological activities such as bronchodilator, antiemetic, diuretic, analgesic, anti-inflammatory, and antirheumatic effects [3, 4]. Different experimental evidences have suggested that the anti-inflammatory properties of some species of genus* Pistacia* are due to the enzymatic inhibition of cyclooxygenase and lipoxygenase. These effects seem to be related to the presence of terpenoids and flavonoids [5, 6]. On this basis, this study was focused on the isolation and characterization of the constituent(s) from a methanolic extract of* P. integerrima* barks and it was also oriented to evaluate its biological activity by a classical* in vivo* and* in silico* approach.

## 2. Materials and Methods

### 2.1. Plant Material

*P. integerrima* was collected from Murree Hills (Pakistan) and it was successively identified by Prof. Rashid A. (Department of Botany, University of Peshawar, Pakistan). A voucher specimen (number 20037) was deposited at Department of Botany, University of Peshawar, Pakistan.

### 2.2. Extraction and Isolation

The barks (8.9 kg) were shade-dried at room temperature, grounded into powder, and subsequently extracted thrice with methanol at room temperature, giving 387 g of residue. The methanol extract was dissolved in water and successively extracted with* n*-hexane (44.2 g), chloroform (98.3 g), ethyl acetate (49.4 g), and* n-*butanol (66.9 g). An aliquot (10.2 g) of the chloroform extract was chromatographed on a silica gel column, eluting with* n*-hexane and ethyl acetate mixtures of increasing polarity. Two hundred and fifty-five fractions were eluted and combined in 10 major fractions (RF-1 to RF-10), according to their TLC similarity. From RF-3 (61.2 mg; eluted with* n*-hexane-EtOAc, 82 : 18) compound** 1** was obtained as colorless crystals which were separated from the solution by decantation. The crystals were recrystallized with appropriate solvents (*n*-hexane-acetone, 4 : 1). The structural elucidation of the isolated compound was performed by spectroscopic methods (^1^H-NMR,^13^C-NMR, HMBC, HMQC, NOESY, COSY, HREI-MS, and IR). Spectra were obtained on a Vector 22 (Bruker) Fourier transform infrared (FTIR) spectrometer, employing KBr windows with CH_2_Cl_2_ as the solvent against an air background. ^1^H-NMR (600 MHz) and ^13^C-NMR (125 MHz) spectra were registered on a Bruker Avance spectrometer. The 2D-NMR spectra were obtained on a Bruker Avance NMR spectrometer. Mass spectral information (EI and HR-EI-MS) was recorded on Jeol-JMS-HX-110 mass spectrometer and calculated in electron impact mode on Finnigan MAT-312 and MAT-95 XP; ions were given in *m*/*z* (%). Melting points of compound** 1 **were determined in glass capillaries tubes by Bicote melting point apparatus (Bibby Scientific limited, UK) and the UV spectra were measured in chloroform by using UV-visible recording spectrometer Model Hitachi-U-3200 (Japan). The IR spectra were recorded on FT-IR Nicolet 380 (Thermo Scientific, UK) and the single X-rays on Kappa APEXII CCD diffractometer (SADABS; Bruker, 2005).

### 2.3. *In Vivo* Procedures

Male BALB/c mice (25–30 g) were used in all the experiments. Animals were purchased from the Pharmacology Section of the Department of Pharmacy, University of Peshawar (Peshawar, Pakistan). The animals were maintained in standard conditions (22 ± 2°C and light/dark cycles, i.e., 12/12 h) and were fed with standard food and water* ad libitum*. The experimental protocols were approved by the Ethical Committee of the Department of Pharmacy, University of Peshawar (Pakistan). All the experiments were performed in compliance with the rulings of the Institute of Laboratory Animal Resources, Commission on Life Sciences, National Research Council. All efforts were made to minimize animal suffering.

Successively, mice were randomly divided into 9 groups (*n* = 6) and lightly anaesthetized with enflurane 4% mixed in O_2_/N_2_O (1 : 1) atmosphere. A negative control group (group I) was injected with normal saline (10 mL/kg; i.p), whereas positive control groups (II, III, IV, and V) were treated with diclofenac sodium (1.0–30.0 mg/kg; i.p). Groups VI, VII, VIII, and IX received compound** 1** at the doses of 1.0, 3.0, 10.0, and 30.0 mg/kg i.p., respectively. Thereafter, 50 *μ*L of 1% *γ*-carrageenan (dissolved in saline) was injected subcutaneously into the subplantar tissue of the mice right paw, 30 min after administration of drugs. Oedema, measured with a hydropletismometer (Ugo Basile, Milan, Italy), was calculated by subtracting the initial paw volume to the paw volume measured at each time point (1–5 h) and successively reported as percentage (%) of decrease of paw volume, as previously described [7, 8].

In acetic acid-induced writhing test, animals were divided into 9 experimental groups (*n* = 6) and successively withdrawn from food 2 h before the experimental section. Group I (control group) was injected with saline (10 mL/kg, i.p.). Groups II, III, IV, and V received diclofenac sodium as standard drug (1.0–30.0 mg/kg, i.p.), while groups VI, VII, VIII, and IX were treated with 1.0, 3.0, 10.0, and 30.0 mg/kg i.p. of** 1**, respectively. After 30 min, mice were treated intraperitoneally (i.p.) with 0.1 mL of a 1% solution of acetic acid. The number of abdominal constrictions (writhings) was counted 5 min after the acetic acid injection for 10 minutes of observation, as previously described [9]. Percentage (%) of anti-inflammatory effect was calculated using the following formula: % anti-inflammatory effect = number of writhings in tested animals/number of writhings in control animals × 100.

### 2.4. Computational Analysis

The COX-1 and COX-2 receptor amino acid sequence was obtained in the FASTA format from UniProt database (code number: P56476) followed by BLAST against PDB database for template selection [10]. The BLAST is commonly used to identify similar homologous structures which can provide template for homology model building [11]. The crystal structures of COX-1 and COX-2 receptor were obtained as the best hit according to their sequence identity. Obtaining the target and template sequence, successively the alignment was carried out by BioEdit sequence alignment editor software [12]. The 3D structures of COX receptors were generated by using MODELLER 9.12. The energy refinement process was carried out through Swiss PDB viewer v4.1.0 software [13]. Energy minimization was performed (500 steps of steepest descent followed by 1000 steps of conjugate gradient) without assigning any constraint [14]. All the residues adopt a stable conformation by avoiding steric hindrance. The refine 3D structures were validated by ProSA and Procheck online servers and the best model was selected for the docking studies [15]. The iGEMDOCK v2.1 software was used for the docking studies of COX-1 and COX-2 with compound** 1**. The 2D structure of the ligands was drawn through ChemDraw software and saved in mol format. The 2D structures were converted into 3D followed by reduction and minimization through Avogadro software [16, 17]. The iGEMDOCK software was implemented with generic evolutionary algorithm (GA) to carry out automated molecular dockings. AutoDock Vina software was also used for the docking analysis. The software can work through AutoDock Tools (ADT) or Pyrex tools [18]. The macromolecules were cleaned from water residues and Gasteiger charges were calculated. The ligand molecules were prepared in ChemDraw software and Avogadro software and were saved in mol2 format. The docking procedure was calibrated by already cocrystallized ligand. The ligands and macromolecules were uploaded in the Pyrex tool [19]. Finally, the receptor and ligand files were converted into pdbqt format.

### 2.5. Statistical Analysis

The results obtained were expressed as the mean ± S.E.M for the raw data or as the mean ± S.E.M of the percentage of the vehicle response, where each animal acted as its own control. For statistical analysis, one-way analysis of variance (ANOVA) was followed by Bonferroni correction for normally distributed data or by Dunnett's for nonparametric data in order to evaluate specific differences between individual groups. In some case, one sample *t*-test was used to evaluate significance against the hypothetical zero value. The ED_50_ value for the anti-inflammatory effect of compound** 1 **accompanied by its respective 95% confidence limits was determined by linear regression from individual experiments using GraphPad Prism 5.0 software (San Diego, CA, USA). Values were considered to be significant at *p* ≤ 0.05.

## 3. Results and Discussion 

The continuing search for new anti-inflammatory agents is due to the complexity of the inflammatory process and its role in host defense. However, the progress achieved in understanding the mechanisms involved in the inflammatory response has made the identification of new targets possible, paving the way for the search for new compounds with potential therapeutic effects on acute or chronic inflammatory diseases [20–23].

Most drug discovery is focused on the search for bioactive compounds obtained from natural sources and many drugs used today for the treatment of several diseases have been developed from natural products. In this context, terpenes, which comprise a very large family of natural products containing more than 50,000 structurally diverse compounds, have recently attracted the attention for their potential anti-inflammatory activities [24–26].

**Figure 1:**
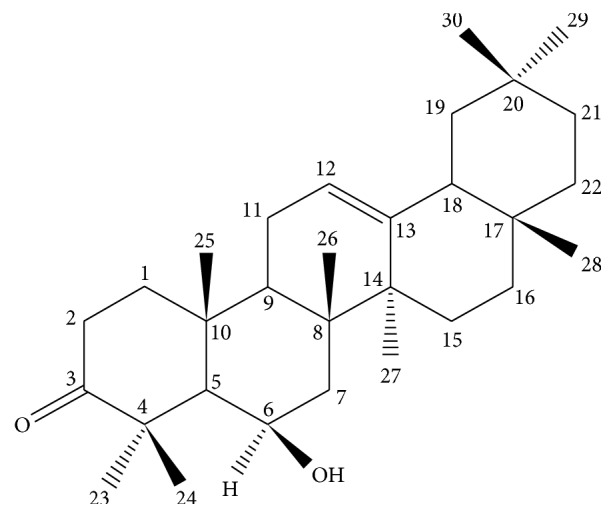
Structure of daturaolone (compound** 1**).

Here, for the first time, we have isolated from a methanol extract of* P. integerrima* barks a pentacyclic triterpenoid derivative identified as daturaolone (compound** 1**). The molecular formula of compound** 1** was determined as C_30_H_48_O_2_ (Figure 1) by EIMS (*m*/*z*; 440.37; calcd. 440.3710) and NMR data. The assignment of protons and carbons was carried out by HMBC, HSQC, and ^1^H-^1^H-COSY experiments (Supplementary Material available online at http://dx.doi.org/10.1155/2016/4098686). Successively, the structure of compound** 1** was confirmed by comparing its NMR and physical and X-ray crystallographic data with those already reported in literature [27].

**Figure 2:**
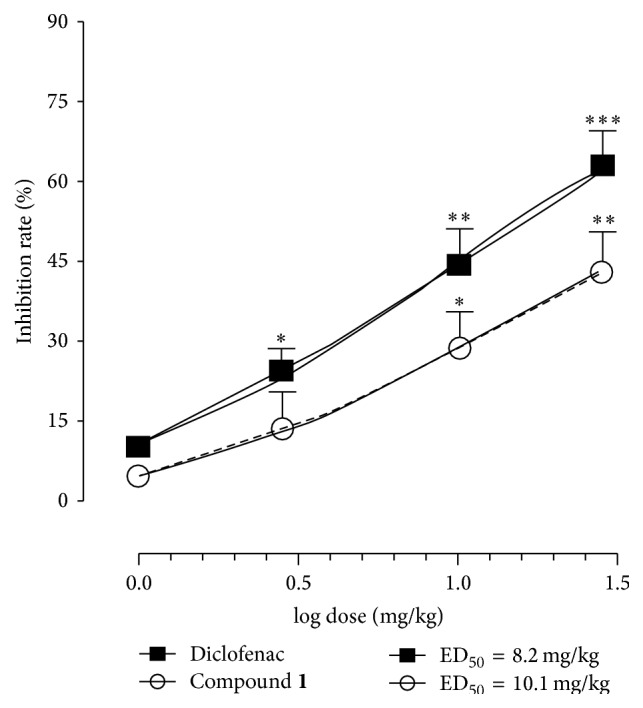
Effect of compound** 1** on carrageenan-induced paw oedema. Compound** 1** (1.0–30.0 mg/kg) or diclofenac (1.0–30.0 mg/kg) was administered intraperitoneally (i.p.) 30 min before the subcutaneous injection of 50 *μ*L of 1% carrageenan and paw swelling measured at 4 h. Values reported as percentage (%) of inhibition of paw oedema are expressed as log dose (mg/kg) ± SEM (*n*  =  6). ^*∗*^ *p* < 0.05, ^*∗∗*^ *p* < 0.01, and ^*∗∗∗*^ *p* < 0.001.

In order to evaluate the potential anti-inflammatory activity of compound** 1**, the carrageenan-induced mouse paw oedema model was employed. The intraplantar injection of carrageenan in mice leads to paw oedema, the first phase of which results from the early (1-2 hrs) release of histamine, serotonin, and kinins followed by a second phase (3-4 hrs) characterized by the production of prostaglandins and oxygen-derived free radicals and the production of inducible cyclooxygenase (COX-2). Only the presence of persistent stimuli or dysregulation of the resolution phase tempts the recruitment of local neutrophil activation and lymphocytes infiltration. For these reasons, carrageenan-induced paw oedema has been shown to be a useful model for the study of inflammation and for the evaluation of anti-inflammatory profiles of various drugs [28–30]. Results of the carrageenan-induced paw oedema showed the dose dependent anti-inflammatory profile of compound** 1** in all time-course (1–5 hrs). The maximum anti-inflammatory effect was observed at 4 h, where compound** 1** significantly (*p* < 0.05 and *p* < 0.01 for 10 and 30 mg/kg, resp.) displayed its anti-inflammatory profile (ED_50_ = 10.1 mg/kg) (Figure 2). More remarkable anti-inflammatory effects were observed after the administration of the reference drug, diclofenac (1.0–30.0 mg/kg; ED_50_ = 8.2 mg/kg) (Figure 2). The results observed at 4 h in the carrageenan-induced paw oedema confirmed the hypothesis that compound** 1** could act by a mechanism related to the inhibition of COXs [31, 32].

**Figure 3:**
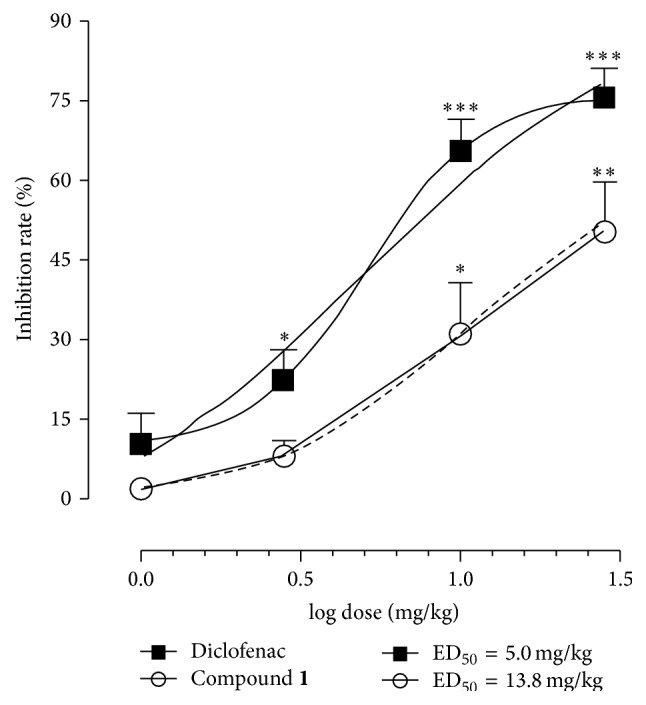
Effect of compound** 1** and diclofenac on acetic acid-induced writhing responses in mice. Saline (10 mL/kg), compound** 1** (1.0, 3.0, 10.0, and 30.0 mg/kg), or diclofenac (1.0, 3.0, 10.0, and 30.0 mg/kg) was administrated intraperitoneally (i.p.) 30 min before acetic acid injection (0.1 mL; i.p.). Values reported as percentage (%) of inhibition of writhings are expressed as log dose (mg/kg) ± SEM (*n*  =  6). ^*∗*^ *p* < 0.05, ^*∗∗*^ *p* < 0.01, and ^*∗∗∗*^ *p* < 0.001.

To investigate the effect of compound** 1** in another* in vivo* model of inflammation, the writhing test was employed. The acetic acid-induced abdominal writhing is a widely used model to detect the anti-inflammatory and analgesic potential of tested compound(s). In this test, the pain induction is due to the release of arachidonic acid and cyclooxygenase products. Drugs able to inhibit the writhing test (e.g., nonsteroidal anti-inflammatory drugs (NSAIDs)) possess analgesic and anti-inflammatory effects associated with the modulation of prostaglandins [32]. Anti-inflammatory effects of** 1** on writhing test are shown in Figure 3. Compound** 1** administered i.p. at different doses (1.0, 3.0, 10.0, and 30.0 mg/kg) exhibited significant (^*∗*^ *p* < 0.05 and ^*∗∗*^ *p* < 0.01) inhibition of writhings with an extent of 31.00 ± 9.53% and 50.33 ± 8.95% at 10.0 and 30.0 mg/kg, respectively. No significant effects were observed at dose of 1.0 and 3.0 mg/kg (ED_50_ = 13.8 mg/kg). Maximal inhibition of the writhing response was slightly lower compared to inhibition evoked by diclofenac sodium at a dose of 30.0 mg/kg (75.33 ± 5.48%; ^*∗∗∗*^ *p* < 0.001). Moreover, the reference drug displayed a significant anti-inflammatory effect even at dose of 3.0 mg/kg (21.67 ± 6.17%; ^*∗*^ *p* < 0.05) and 10.0 mg/kg (65.33 ± 7.78%; ^*∗∗∗*^ *p* < 0.001) (ED_50_ = 5.0 mg/kg).

**Figure 4:**
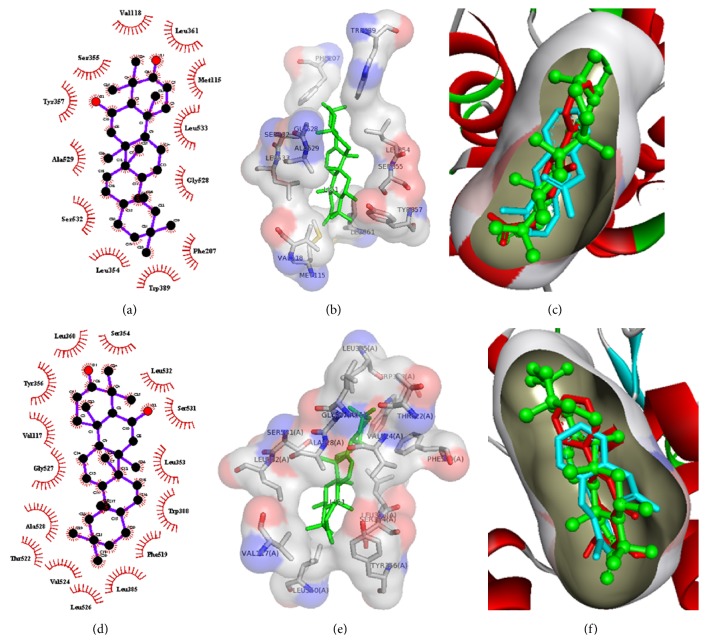
2D and 3D model of compound** 1** in the binding site of COX-1 ((a) and (d)) and COX-2 ((b) and (e)). Half-moon indicates the hydrophobic interactions. ((c) and (f)) Superimposition of compound** 1** (colored by green) and diclofenac (colored by cyan) in the binding site of COX-1 (c) and COX-2 (f) enzyme (colored by red).

Successively, in order to validate the* in vivo* anti-inflammatory activity of compound** 1**, we investigated its mechanism of action from the viewpoint of* in silico* system. By docking studies, we have evaluated the interaction of compound** 1** with the binding site of COXs enzymes. The analysis of receptor ligand complex based on the hydrogen bond interaction and hydrophobic interaction shows that compound** 1** displayed a remarkable interaction with COX-1, whereas it interacts weakly with COX-2 binding site.  Figure 4 shows that compound** 1** establishes twelve hydrophobic contacts with COX-1 receptor formed by Meth115, Val118, Phe207, Leu354, Ser355, Tyr357, Leu361, Trp389, Gly528, Ala529, Ser532, and Leu533 (Figures 4(a) and 4(b)) and fifteen contacts with COX-2 receptor formed by Val117, Leu353, Tyr356, Leu360, Ser354, Leu385, Trp388, Phe519, Thr522, Val524, Leu526, Gly527, Ala528, Leu532, and Ser531 (Figures 4(d) and 4(e)). However, no hydrogen bonding was observed. In order to rationalize the binding mode of compound** 1**, we have used the crystal structure of COXs in complex with diclofenac, a nonspecific ligand of this receptor. In particular, the docking results of** 1** on COX-1 showed a binding energy of −7.0 kcal/mol and a total energy of −90 kcal/mol, whereas these energy values were of −7.5 kcal/mol and −95 kcal/mol for COX-2 (Figures 4(c) and 4(f) for COX-1 and COX-2, resp.). The interaction energies of compound** 1** with COX-1s receptor, compared to standard ligand, diclofenac (−5.7 kcal/mol and −80 kcal/mol in terms of binding and total energy, resp.), were most likely sufficient to justify the selective anti-inflammatory (COX-1 mediated) activity of the tested compound.

## 4. Conclusions

In our continuing program on the chemical and pharmacological characterization of natural compounds, a triterpenoid identified as daturaolone (compound** 1**) was isolated from* Pistacia integerrima*. Compound** 1** displayed an interesting anti-inflammatory activity compared to diclofenac as demonstrated in classical* in vivo* models of inflammation. Moreover, by* in silico* studies, we have also established that this compound displayed a COX-1 inhibitory activity. However, further studies are needed to better clarify this anti-inflammatory activity and to discern its peripheral and/or central effect.

## Supplementary Material

The structural elucidation of the isolated compound was performed by spectroscopic methods (^1^H-NMR, ^13^C-NMR, HMBC, HMQC, NOESY, COSY, HREI-MS, and IR). Spectra were obtained on a Vector 22 (Bruker) Fourier transform infrared (FTIR) spectrometer, employing KBr windows with CH_2_Cl_2_ as the solvent against an air background. ^1^H-NMR (600 MHz) and ^13^C-NMR (125 MHz) spectra were registered on a Bruker Avance spectrometer. The 2D-NMR spectra were obtained on a Bruker Avance NMR spectrometer. Mass spectral information (EI and HR-EI-MS) was recorded on Jeol-JMS-HX-110 mass spectrometer and calculated in electron impact mode on Finnigan MAT-312 and MAT-95 XP; ions were given in m/z (%). Melting points of compound 1 were determined in glass capillaries tubes by Bicote melting point apparatus (Bibby Scientific limited, UK) and the UV spectra were measured in chloroform by using UV-visible recording spectrometer Model Hitachi-U-3200 (Japan). The IR spectra were recorded on FT-IR Nicolet 380 (Thermo Scientific, UK) and the single X-rays on Kappa APEXII CCD diffractometer (SADABS; Bruker, 2005).
