# Supplementary material for: Biological Evaluation and Docking Analysis of Daturaolone as Potential Cyclooxygenase Inhibitor
Source: Evid Based Complement Alternat Med. 2016 Mar 2;2016:4098686. doi: 10.1155/2016/4098686 (PMC4793090; doi:10.1155/2016/4098686)
Supplement: Supplementary file 1 — The structural elucidation of the isolated compound was performed by spectroscopic methods (1H-NMR, 13C-NMR, HMBC, HMQC, NOESY, COSY, HREI-MS, and IR). Spectra were obtained on a Vector 22 (Bruker) Fourier transform infrared (FTIR) spectrometer, employing KBr windows with CH2Cl2 as the solvent against an air background. 1H-NMR (600 MHz) and 13C-NMR (125 MHz) spectra were registered on a Bruker Avance spectrometer. The 2D-NMR spectra were obtained on a Bruker Avance NMR spectrometer. Mass spectral information (EI and HR-EI-MS) was recorded on Jeol-JMS-HX-110 mass spectrometer and calculated in electron impact mode on Finnigan MAT-312 and MAT-95 XP; ions were given in m/z (%). Melting points of compound 1 were determined in glass capillaries tubes by Bicote melting point apparatus (Bibby Scientific limited, UK) and the UV spectra were measured in chloroform by using UV-visible recording spectrometer Model Hitachi-U-3200 (Japan). The IR spectra were recorded on FT-IR Nicolet 380 (Thermo Scientific, UK) and the single X-rays on Kappa APEXII CCD diffractometer (SADABS; Bruker, 2005). [file 4098686.f1.pdf]

ICCBS(HEJ-LAB-101)

8/21/2013 10:33:30 AM

File: PN
Sample: ABDUR RAUF / DR.BINA S.SIDDIQUI
Instrument: JEOL JMS-600H
Inlet: Direct Probe

Date Run: 08-21-2013 (Time Run: 10:20:33)

Ionization mode: EI+

Run By: HEJ (ICCBS]

Scan: 22
Base: m/z 218; 21.5%FS TIC: 1906308

R.T.: 1.95

#Ions: 300

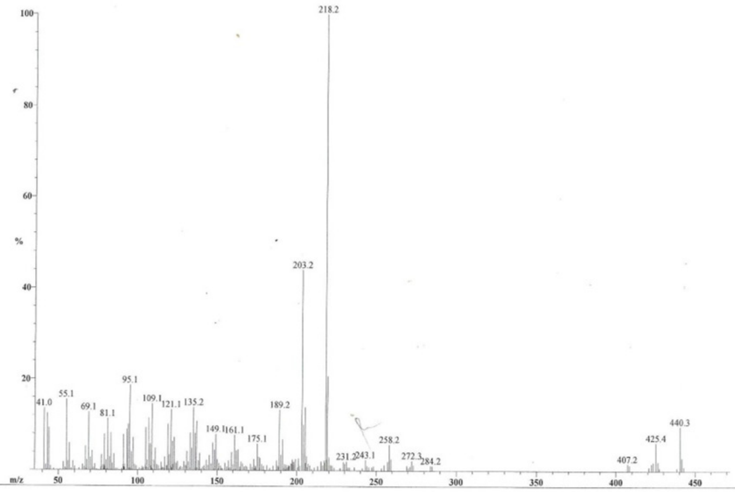

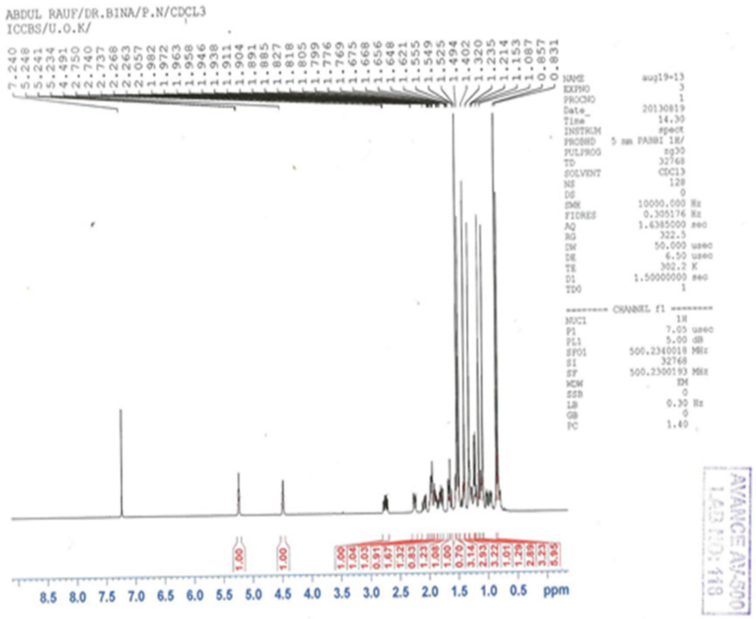
ABDUL RAUF/DR.BINA/P.N/CDCL3
ICCBS/U.O.K/
7.240
5.248
5.241
5.234
4.491
2.750
2.740
2.737
2.268
2.263
2.057
1.982
1.972
1.963
1.958
1.946
1.938
1.911
1.904
1.891
1.885
1.827
1.818
1.805
1.799
1.776
1.769
1.675
1.668
1.656
1.648
1.621
1.555
1.549
1.525
1.494
1.402
1.320
1.235
1.214
1.153
1.087
0.857
0.831
NAME aug19-13
EXPNO 3
PROCNO 1
Date_ 20130819
Time 14.30
INSTRUM spect
PROBHD 5 mm PABBI 1H/
PULPROG zg30
TD 32768
SOLVENT CDCl3
NS 128
DS 0
SWH 10000.000 Hz
FIDRES 0.305176 Hz
AQ 1.6385000 sec
RG 322.5
DW 50.000 usec
DE 6.50 usec
TE 302.2 K
D1 1.50000000 sec
TD0 1
======== CHANNEL f1 ========
NUC1 1H
P1 7.05 usec
PL1 5.00 dB
SFO1 500.2340018 MHz
SI 32768
SF 500.2300193 MHz
WDW EM
SSB 0
LB 0.30 Hz
GB 0
PC 1.40
1.00
1.00
8.5 8.0 7.5 7.0 6.5 6.0 5.5 5.0 4.5 4.0 3.5 3.0 2.5 2.0 1.5 1.0 0.5 ppm
AVANCE AV-500
LAB NO: 118

ABDUR RAUF/DR.BINA/PN/CDCL3
BB

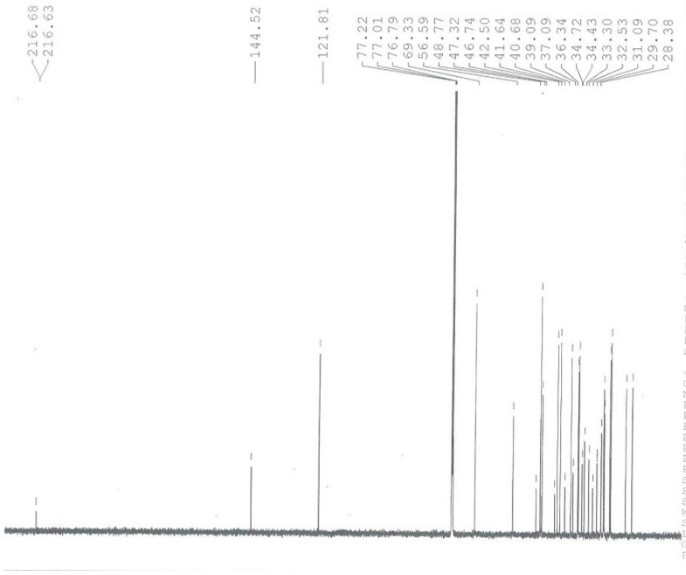

AVANCE AV 600
LAB. No. 108
LC - NMR

NAME	aug26-13	
EXPNO	6	
PROCNO	1	
Date_	20130827	
Time	12.17	
INSTRUM	spect	
PROBHD	5 mm PATXI 1H-	
PULPROG	zgpg	
TD	65536	
SOLVENT	CDCl3	
NS	20480	
DS	4	
SWH	35971.223	Hz
FIDRES	0.548877	Hz
AQ	0.9110143	sec
RG	32768	
DW	13.900	usec
DE	6.50	usec
TE	299.9	K
D1	2.00000000	sec
D11	0.03000000	sec
TD0	20	
======== CHANNEL f1 ========		
NUC1	13C	
P1	11.60	usec
PL1	-4.00	dB
PL1W	135.65582275	W
SFO1	150.8950149	MHz
======== CHANNEL f2 ========		
CPDPRG2	waltz16	
NUC2	1H	
PCPD2	100.00	usec
PL2	0.00	dB
PL12	22.50	dB
PL13	28.00	dB
PL2W	14.56894112	W
PL12W	0.08192718	W
PL13W	0.02309022	W
SFO2	600.0336002	MHz
SI	32768	
SF	150.8776640	MHz
WDW	EM	
SSB	0	
LB	1.00	Hz
GB	0	
PC	1.00	

ABDUR RAUF/DR.BINA/PN/CDCL3
dept135

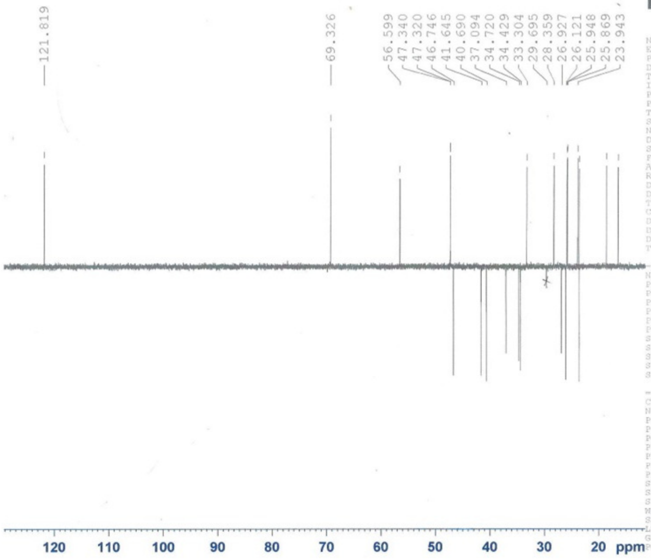

AVANCE AV 600
LAB. No. 106

BRUKER

NAME aug26-13
EXPNO 7
PROCNO 1
Date_ 20130828
Time 4.57
INSTRUM spect
PROBHD 5 mm PATXI 1H-
PULPROG deptsp135
TD 32768
SOLVENT CDCl3
NS 7197
DS 2
SWH 30303.031 Hz
FIDRES 0.924775 Hz
AQ 0.5407385 sec
RG 32768
DW 16.500 usec
DE 6.50 usec
TE 303.3 K
CNST2 145.0000000
D1 1.50000000 sec
D2 0.00344828 sec
D12 0.00002000 sec
TD0 10

======== CHANNEL f1 ========
NUC1 13C
P1 11.60 usec
P12 2000.00 usec
PL0 120.00 dB
PL1 -4.00 dB
PLOW 0.00000000 W
PL1W 135.65582275 W
SFO1 150.8927518 MHz
SP2 1.99 dB
SPNAM2 Crp60comp.4
SPOAL2 0.500
SPOFFS2 0.00 Hz

======== CHANNEL f2 ========
CPDPRG2 waltz16
NUC2 1H
P3 7.40 usec
P4 14.80 usec
PCPD2 100.00 usec
PL2 0.00 dB
PL12 22.50 dB
PL2W 14.56894112 W
PL12W 0.08192718 W
SFO2 600.0330002 MHz
SI 32768
SF 150.8776640 MHz
WDW EM
SSB 0
LB 0.50 Hz
GB 0
PC 1.40

ABDUR RAUF/DR.BINA/PN/CDCL3
hmbc

AVANCE AV 600
LAB. No. 198
LC NMR
BRUKER

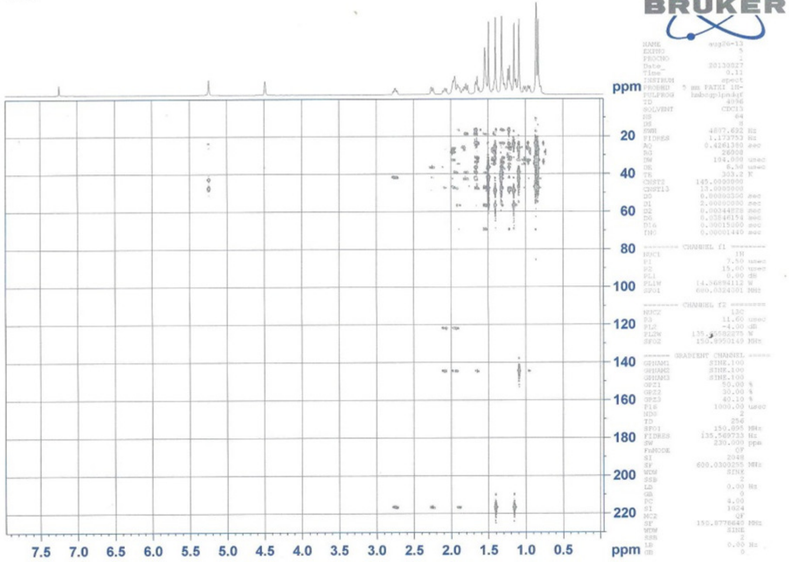

ABDUR RAUF/DR.BINA/PN/CDCL3
hsqc

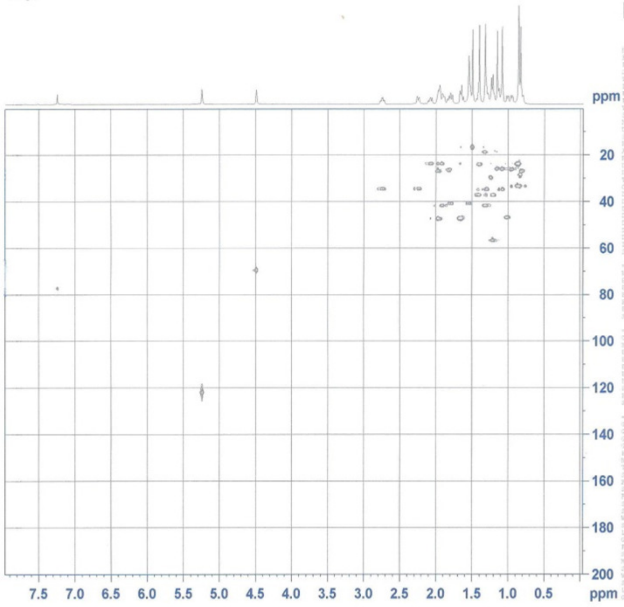

ABDUR RAUF/DR.BINA/PN/CDCL3
noesy

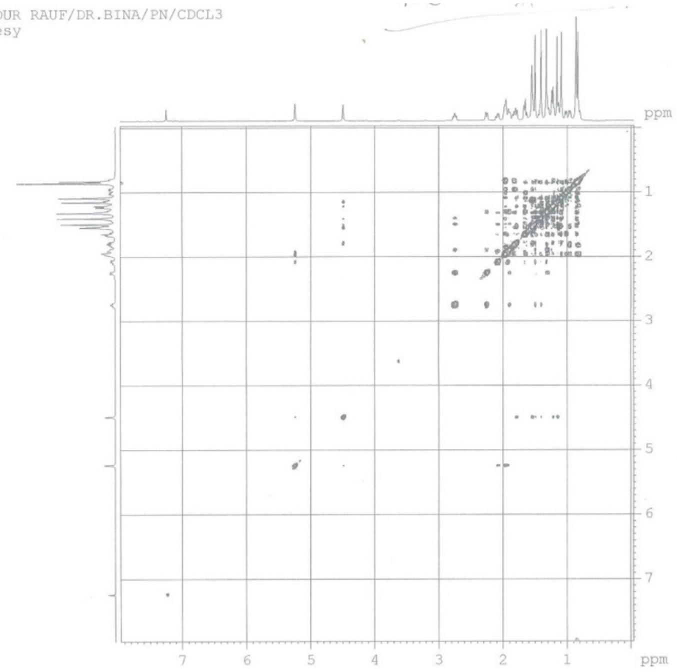

NAME	aug26-13	
EXPNO	3	
PROCNO	1	
Date_	20130826	
Time	15.51	
INSTRUM	spect	
PROBHD	5 mm PATXI 1H-	
PULPROG	noesygpph	
TD	2048	
SOLVENT	CDCl3	
NS	16	
DS	4	
SWH	4807.692	Hz
FIDRES	2.347506	Hz
AQ	0.2131460	sec
RG	812.7	
DW	104.000	usec
DE	6.50	usec
TE	300.1	K
D0	0.00009445	sec
D1	2.00000000	sec
D8	0.80000001	sec
D16	0.00015000	sec
IN0	0.00020800	sec

======== CHANNEL f1 ========

NUC1	1H	
P1	7.50	usec
P2	15.00	usec
PL1	0.00	dB
PL1W	14.56894112	W
SFO1	600.0324001	MHz

====== GRADIENT CHANNEL =====

GPNAM1	SINE.100	
GPNAM2	SINE.100	
GPZ1	40.00	%
GPZ2	-40.00	%
P16	2000.00	usec
ND0	1	
TD	256	
SFO1	600.0324	MHz
FIDRES	18.780046	Hz
SW	8.012	ppm
FnMODE	States-TPPI	
SI	1024	
SF	600.0300255	MHz
WDW	SINE	
SSB	2	
LB	0.00	Hz
GB	0	
PC	4.00	
SI	512	
MC2	States-TPPI	
SF	600.0300255	MHz
WDW	SINE	
SSB	2	
LB	0.00	Hz
GB	0	

ABDUR RAUF/DR.BINA/PN/CDCL3
cosy

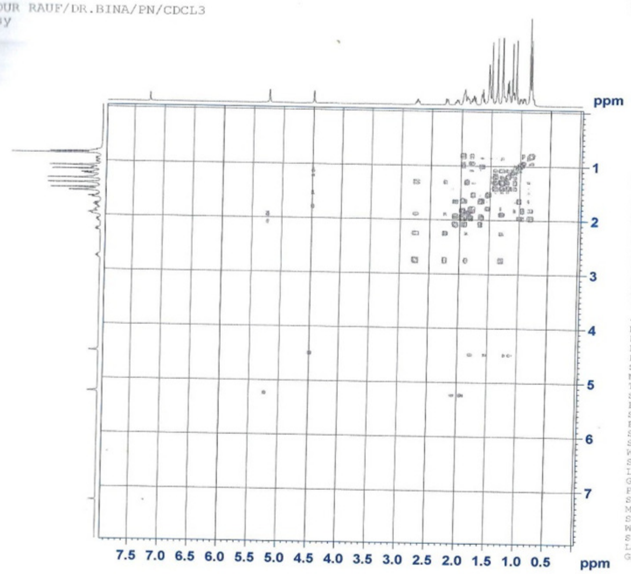

AVANCE AV 600
LAS. No. 108
LC - NMR

NAME	aug26-13
EXPNO	2
PROCNO	1
Date_	20130826
Time	14.33
INSTRUM	spect
PROBHD	5 mm PATXI 1H-
PULPROG	cosydfqf
TD	2048
SOLVENT	CDCl3
NS	8
DS	4
SWH	4807.692 Hz
FIDRES	2.347506 Hz
AQ	0.2131460 sec
RG	90.5
DW	104.000 usec
DE	6.50 usec
TE	300.3 K
D0	0.00000300 sec
D1	2.00000000 sec
D13	0.00000400 sec
D20	0.00000200 sec
IN0	0.00020800 sec

======== CHANNEL f1 ========

NUC1	1H
P1	7.50 usec
PL1	0.00 dB
PL1W	14.56894112 W
SFO1	600.0324001 MHz
ND0	1
TD	256
SFO1	600.0324 MHz
FIDRES	18.780046 Hz
SW	8.012 ppm
FnMODE	QF
SI	1024
SF	600.0300255 MHz
WDW	QSINE
SSB	0
LB	0.00 Hz
GB	0
PC	4.00
SI	1024
MC2	QF
SF	600.0300255 MHz
WDW	QSINE
SSB	0
LB	0.00 Hz
GB	0

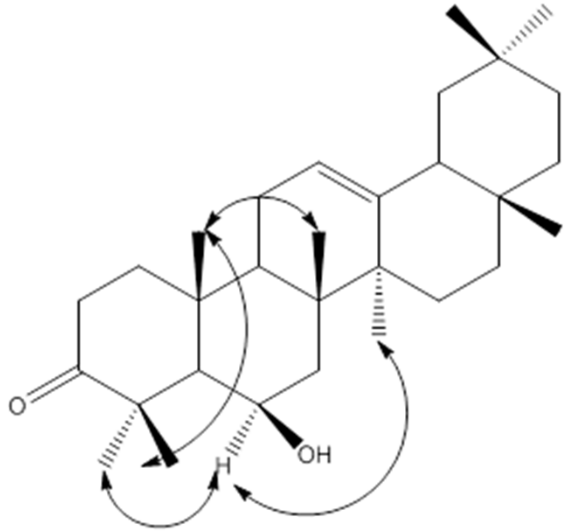
O
H
OH

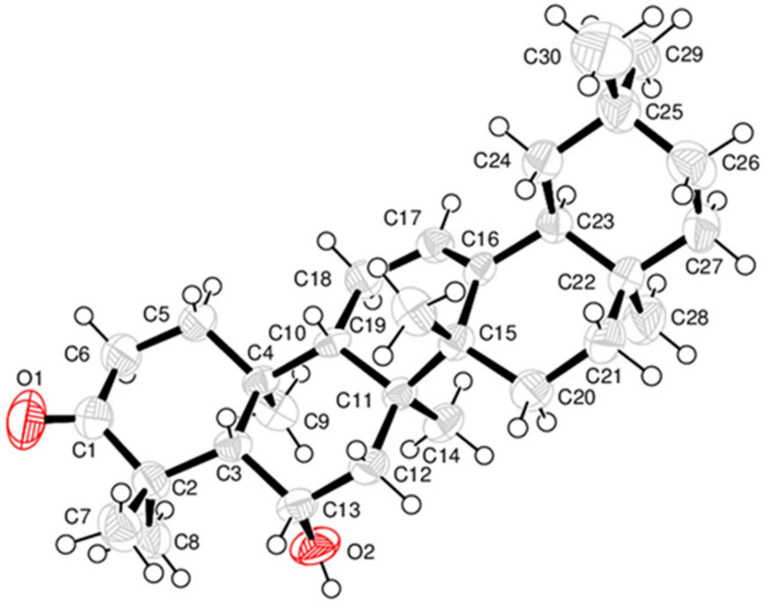
C30
C29
C25
C24
C26
C17
C23
C16
C18
C22
C27
C28
C5
C10
C19
C15
C6
C4
C21
O1
C11
C20
C9
C1
C14
C3
C12
C2
C13
C7
C8
O2
